# Supplementary material for: Closed and open structures of the eukaryotic magnesium channel Mrs2 reveal the auto-ligand-gating regulation mechanism
Source: Nat Struct Mol Biol. 2024 Nov 28;32(3):491–501. doi: 10.1038/s41594-024-01432-1 (PMC11919701; doi:10.1038/s41594-024-01432-1)
Supplement: Supplementary file 1 — Supplementary Figs. 1–3 and Table 1. [file 41594_2024_1432_MOESM1_ESM.pdf]

nature structural & molecular biology

Supplementary information https://doi.org/10.1038/s41594-024-01432-1

Closed and open structures of the eukaryotic magnesium channel Mrs2 reveal the auto-ligand-gating regulation mechanism

In the format provided by the authors and unedited

Supplementary information

Supplementary Discussion

Supplementary Fig. 1

Supplymentary Fig. 2

Supplementary Fig. 3

Supplementary Fig. 4

Supplementary Table 1

Supplementary References

Supplementary Discussion

In this work, we propose a Mg^{2+} auto-ligand-regulated permeation mechanism (Fig. 6). As indicated in the main manuscript, fully hydrated Mg^{2+} from the intermembrane space are attracted and concentrated by two layers of negatively charged residues in the pentamer, the E449 layer and the E450 layer. E449 that forms the inner ring and N443 may well be involved in the water stripping, as Mg^{2+} cannot be fully hydrated as it binds to site U. Next, the ion is transferred to the S site formed by the GMN-filter that defines the entire CorA/Mrs2 family of proteins. This represents the narrowest point of the open channel and thus is likely important to establish specificity, as supported by inactivating mutations (Fig. 2g and 5). Nevertheless, the site is continuously present in the open and closed structures, and hence a role in preventing (back)flow cannot be excluded. Passage of partially hydrated Mg^{2+} towards to matrix is then facilitated by hydrophilic main- and/or sidechains, some of which are conserved such as T427. We note such permeation would be reminiscent of the conductance of partially dehydrated Mg^{2+} proposed for TRPM7, via a tetrameric filter marked by Phe-Gly-Glu and Tyr residues [1,2]. Conversely, permeation of fully hydrated Mg^{2+} has been suggested for dimeric MgtE/SLC41 carriers via a conserved, ion-selecting aspartate [3], while CorB/CorC/CNNM/ACDP transporters recognize fully dehydrated Mg^{2+} in the transmembrane domain [4,5]. In Mrs2, ion exit to the matrix is orchestrated by a set of pore-lining positively charged and hydrophobic residues, in particular R406, R413 and M417 in CtMrs2. The configuration of these gates is in-turn controlled by the soluble domains that are present in the matrix, with considerable effects on these constrictions. The flux is likely energized by the mitochondrial membrane potential (higher pH in the mitochondrial matrix) and the Mg^{2+} gradient across the inner membrane [6,7].

Interestingly, the available structures of hMrs2 generally agree with the here proposed notion of permeation and regulation, detecting a similar homo-pentameric architecture overall, with a long ion-conduction pathway. Furthermore, hMrs2 and CtMrs2 share common features with conserved negatively charged residues for Mg^{2+} uptake, an invariant GMN selectivity filter, a hydrophilic pore, gating residues (with rings of Met-Arg-Asp, and then an amino acid with a large sidechain) and finally the (M1 and M4) Mg^{2+} sensor sites in the soluble domains, shown here to be important for the function. This all points towards the Mrs2 family of proteins operating using similar molecular principles. Indeed, for hMrs2 Mg^{2+} remains at the S (of the GMN-selectivity filter) and P2 (of the pore) sites following treatment with EDTA (PDB-IDs: 8TUP, 8IP4 and 8IP5), which largely is consistent with our observations of the ion at the S, P1 and P2 sites. We interpret this as if the sites of the ion-conductance pathway have high-affinity

for Mg^{2+}, perhaps also contributing to protein stability. However, the hMrs2 structures elucidated in Mg^{2+}-free conditions are not dramatically changed compared to those obtained in the presence of Mg^{2+}, and no major differences are detected at the pore gate, of the matrix-located soluble domains with the M1 and M4 sites, or of the RDLR-motif. This is distinct from our open structure in which the pore gate rings are sufficiently widened to permit passage of Mg^{2+}, and with the M1-M4 Mg^{2+}-sites being replaced by symmetric separation of the soluble domains and the RDLR-motif, hinting at that complete opening has not been triggered for the hMrs2 structures. It is possible this relates to the continuous supplementation of EDTA to the sample we exploited for obtaining the open CtMrs2 structure, but future efforts on hMrs2 will be required to validate this hypothesis.

The here suggested negative feedback regulatory mechanism for Mrs2 resonates with that observed for TRPM7 and MgtE, proteins that serve to provide cellular uptake of the cation. While the molecular bases for the recognition differ, these proteins are also activated and inhibited by low and high intracellular concentrations of Mg^{2+}(-ATP), respectively, through interaction with the soluble domains on the intracellular side [2,8,9]. Conversely, cellular export of Mg^{2+} by CorB/CNNM is oppositely regulated, stimulated and impaired by elevated and reduced levels of the ion, respectively, through interaction with cytoplasmic Mg^{2+}-ATP [10] to the so-called CBS-domains. Collectively, our findings thus shed critical new light on the molecular architecture that permit ion uptake, permeation, gating, and regulation of eukaryotic Mrs2 proteins, results that unify decades of observations on the CorA/Mrs2 family of proteins, and that shed further light on how Mg^{2+} channels and transporters operate.

Supplementary Fig. 1.

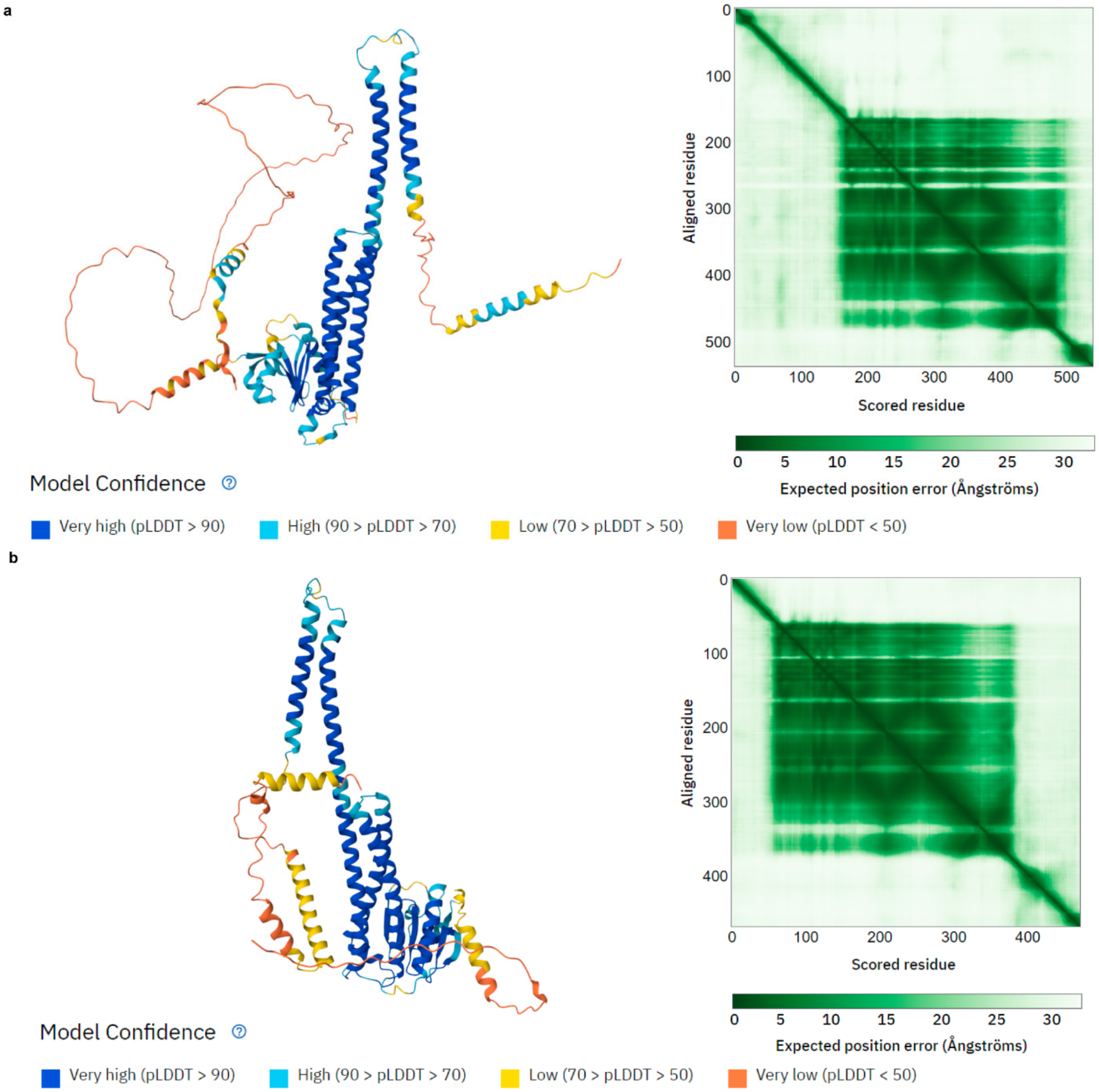

Supplementary Fig. 1. AlphaFold models of CtMrs2 and ScMrs2. a, CtMrs2 AlphaFold model (accession code AF-G0S186-F1) with model confidence score (pLDDT) indicated (left) and predicted aligned error (PAE) plot (right). This model was employed to guide model building. **b,** ScMrs2 AlphaFold model (accession code AF-Q01926-F1) with model confidence score (pLDDT) indicated (left) and predicted aligned error (PAE) plot (right).

Supplementary Fig. 2.

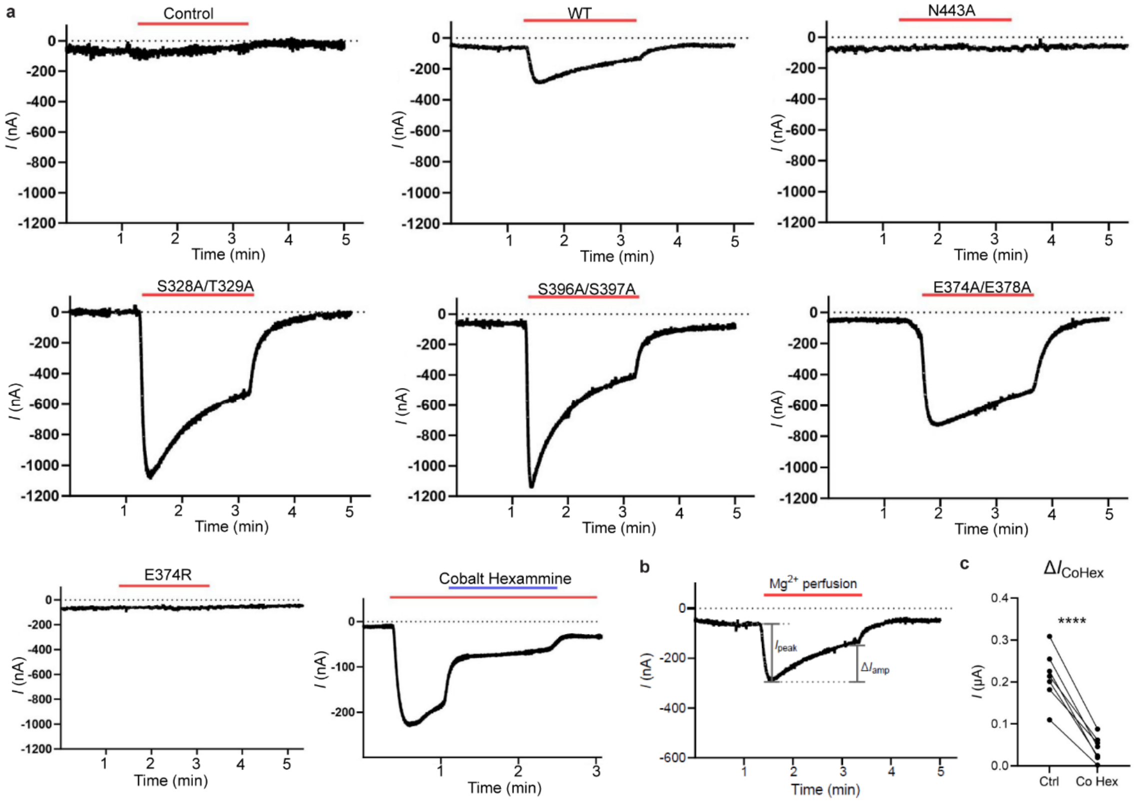

Supplementary Fig. 2. Electrophysiology of CtMrs2. a, Representative Mg^{2+} currents recorded in oocytes expressing WT and mutants (the same as the ones overlayed in Fig. 5c), with the WT activity being inhibited by cobalt hexammine, compared to uninjected controls, and with the N443A mutant of the selectivity filter (known to be non-conductive). Red bars indicate application period of Mg^{2+}-containing recording solution. Blue bar indicates application of 1 mM cobalt hexammine. The holding voltage was -60 mV. **b,** Definition of the spontaneous current decay during Mg^{2+}-perfusion, ΔI_{amp} (%). **c,** Summary plot for cobalt hexammine shows the ability of 1 mM cobalt hexammine to reduce current amplitude. Data is shown for 7 cells (n = 7) expressing WT, where current amplitude without cobalt hexammine (Ctrl) and with cobalt hexammine (Co Hex) is plotted for each cell. A connecting line is included for each cell, to facilitate readout of current amplitude without and with cobalt hexammine for each individual cell. For further details see Methods.

Supplementary Fig. 3.

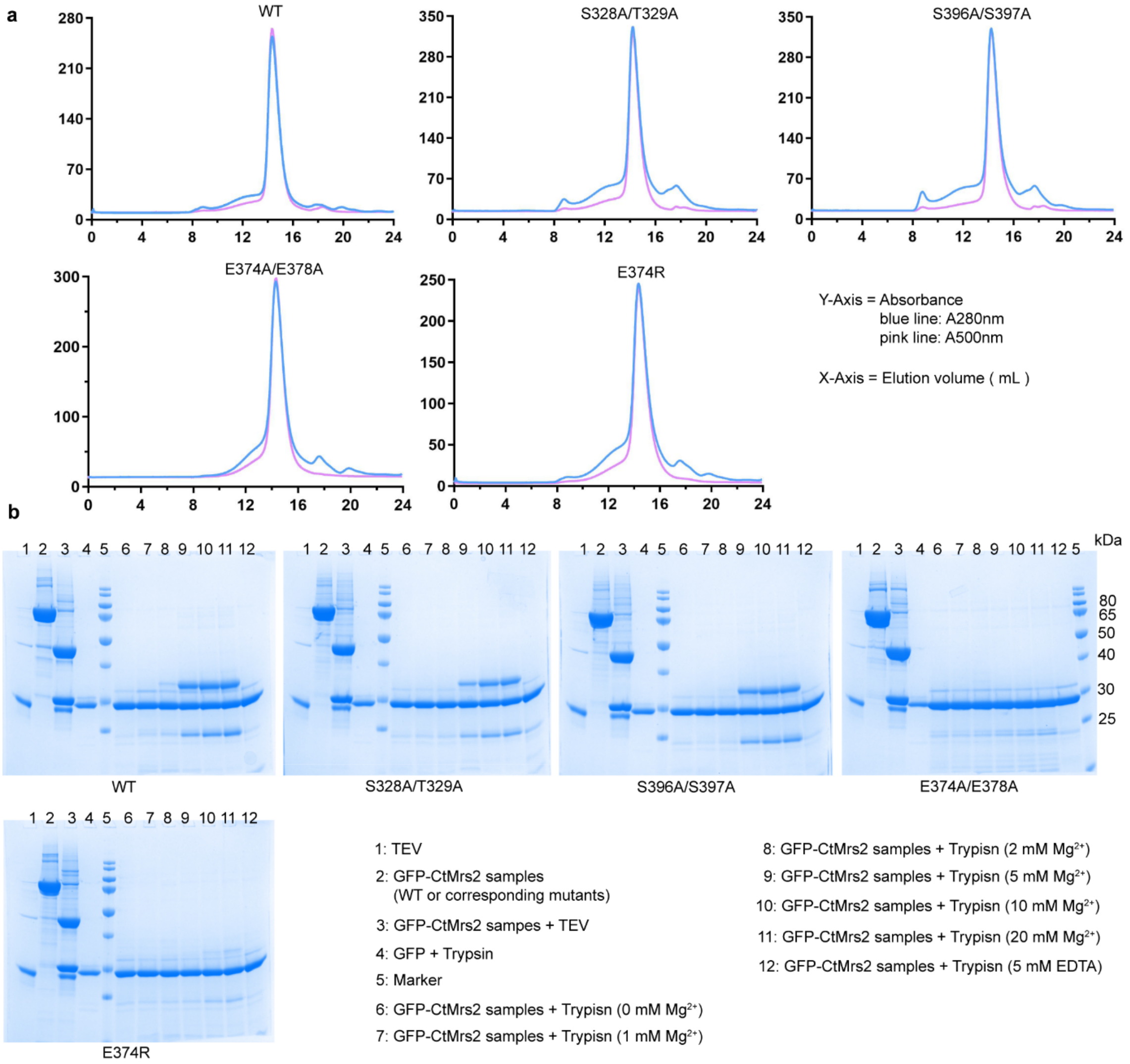

Supplementary Fig. 3. Size-exclusion chromatography profiles and limited proteolysis assay of GFP-CtMrs2 forms. a, Overproduction and purification of the wild-type form were performed at least five independent times, and the other mutants were isolated three times independently. **b,** SDS-PAGE analysis with trypsin treated samples in different conditions (each assay was performed twice independently). The reaction was performed at 37 °C for 1 h. The bands above 30 kDa correspond to CtMrs2 as confirmed by MS. The bands below 30 kDa represent GFP as confirmed by MS.

Supplementary Fig. 4.

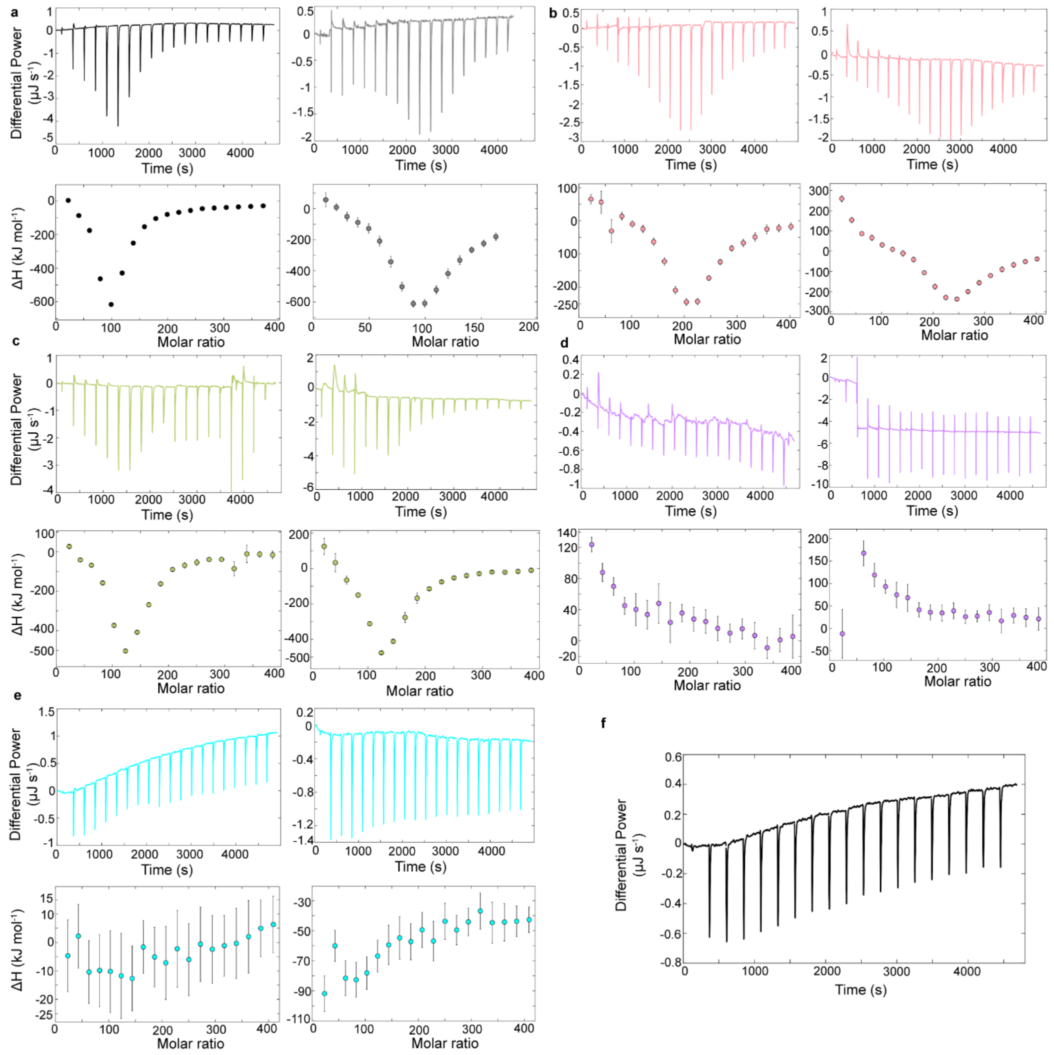

Supplementary Fig. 4. Isothermal titration calorimetry experiments characterizing Mg^{2+}-binding to Mrs2 wild-type (WT) and mutants. Each pair of graphs shows in the upper graph the differential power vsersus time, where differential power is re-referenced to 0 $\mu J{\cdot}s^{-1}$, prior to analysis in NITPIC [68], and in the lower graph show the SVD-reconstructed isotherms from NITPIC analysis as ($\Delta H{\cdot}mol^{-1}$) versus molar ratio of Mg^{2+} to protein monomer. **a,** WT at 50 mM Mg^{2+} (left panel, black), 25 mM Mg^{2+} (right panel, dark grey). **b,** S328A/T329A at 50 mM Mg^{2+} (left panel, pink), 25 mM Mg^{2+} (right panel, pink). **c,** S396A/S397A at 50 mM Mg^{2+} (left panel, light green), 25 mM Mg^{2+} (right panel, light green). **d,** E374A/E378A at 50 mM Mg^{2+} (left panel, purple), 25 mM Mg^{2+} (right panel, purple). **e,** E374R at 50 mM Mg^{2+} (left panel, cyan), 25 mM Mg^{2+} (right panel, cyan). Error bars show 1 SD and are estimated from baseline uncertainties provided by NITPIC as previously described [68]. **f,** control experiment with 50 mM Mg^{2+} in buffer injected into the buffer solution. Each experiment was performed once.

Supplementary Table 1.

1	CtMrs2-22b-F	TCTTTATTTTCAGGGCATGCCTCCGGCATTGAAACCTCTGGC
2	CtMrs2-22b-R	TTAGCAGCCGGATCTCACTATAACTTGTTAAGAGGCCACTTCTTAGCAG
3	Δintron1-F	TGATTGCTAAGTATGGCCTTCTTCCTCGCGATC
4	Δintron1-R	AGAAGGCCATACTTAGCAATCAACTCGCTTTTCGC
5	Δintron2-F	ACGGAGGAAATTATTCGAGCCATCCTGGATGCG
6	Δintron2-R	TGGCTCGAATAATTTCCTCCGTATTCCTGATGCTCG
7	GFP-CtMrs2-F	TTGTATTTTCAATCTATGTCGTCAGGATTCTCCTCGGAG
8	GFP-CtMrs2-R	TTGATATTGGATCATCTATAACTTGTTAAGAGGCCACTTC
9	N443A-F	GGC ATG GCC CTT GAG AAT TTC ATA
10	N443A-R	TCTCAAGGGCCATGCCGTATAG
11	S328A/T329A-F	AGG GTC GCC GCG TTT GAA CAG AAG
12	S328A/T329A-R	TTCAAACGCGGCGACCCTCTTTGAC
13	S396A/S397A-F	CTT GTG GCG GCC ATC AGG AAT ACG
14	S396A/S397A-R	CTGATGGCCGCCACAAGGTTACTC
15	E374R-F	T GAA GTA AGA CTC CTG TTG GAG TC
16	E374R-R	AACAGGAGTCTTACTTCAGTGTG
17	E374A/E378A-F	GTAGCACTCCTGTTGGCGTCGTACCAC
18	E374R/E378A-R	CGACGCCAACAGGAGTGCTACTTCAGTG

Supplementary Table 1. Primers used in this work.

Supplementary References

1. Duan, J. et al. Structure of the mammalian TRPM7, a magnesium channel required during embryonic development. *Proc Natl Acad Sci U S A* **115**, E8201-E8210 (2018).
2. Nadezhdin, K.D. et al. Structural mechanisms of TRPM7 activation and inhibition. *Nat Commun* **14**, 2639 (2023).
3. Takeda, H. et al. Structural basis for ion selectivity revealed by high-resolution crystal structure of Mg2+ channel MgtE. *Nat Commun* **5**, 5374 (2014).
4. Huang, Y. et al. Structural basis for the Mg(2+) recognition and regulation of the CorC Mg(2+) transporter. *Sci Adv* **7**(2021).
5. Chen, Y.S. et al. Crystal structure of an archaeal CorB magnesium transporter. *Nat Commun* **12**, 4028 (2021).
6. Daw, C.C. et al. Lactate Elicits ER-Mitochondrial Mg(2+) Dynamics to Integrate Cellular Metabolism. *Cell* **183**, 474-489 e17 (2020).
7. Kolisek, M. et al. Mrs2p is an essential component of the major electrophoretic Mg2+ influx system in mitochondria. *EMBO J* **22**, 1235-44 (2003).
8. Jin, F. et al. The structure of MgtE in the absence of magnesium provides new insights into channel gating. *PLoS Biol* **19**, e3001231 (2021).
9. Schlingmann, K.P., Waldegger, S., Konrad, M., Chubanov, V. & Gudermann, T. TRPM6 and TRPM7--Gatekeepers of human magnesium metabolism. *Biochim Biophys Acta* **1772**, 813-21 (2007).
10. Chen, Y.S. & Gehring, K. New insights into the structure and function of CNNM proteins. *FEBS J* **290**, 5475-5495 (2023).
