# Supplementary figures and images for: Closed and open structures of the eukaryotic magnesium channel Mrs2 reveal the auto-ligand-gating regulation mechanism
Source: Nat Struct Mol Biol. 2024 Nov 28;32(3):491–501. doi: 10.1038/s41594-024-01432-1 (PMC11919701; doi:10.1038/s41594-024-01432-1)

Fig. 2g

YPD

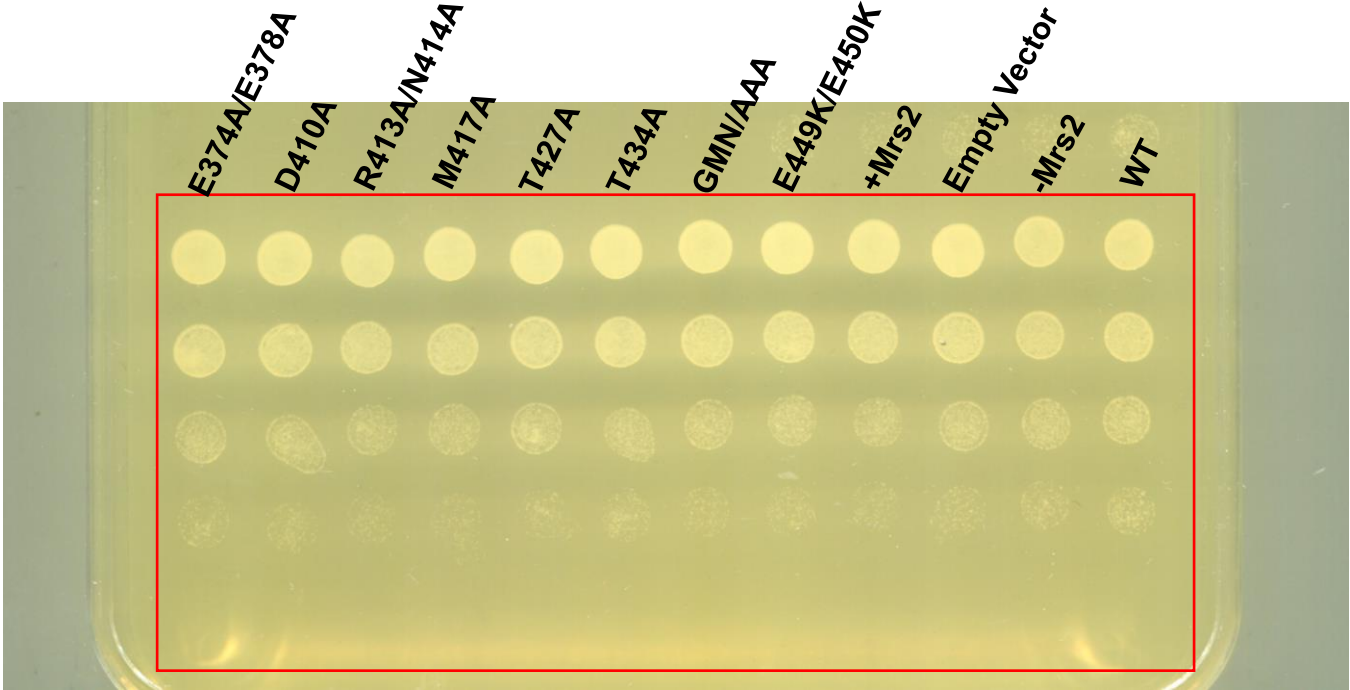

YPG

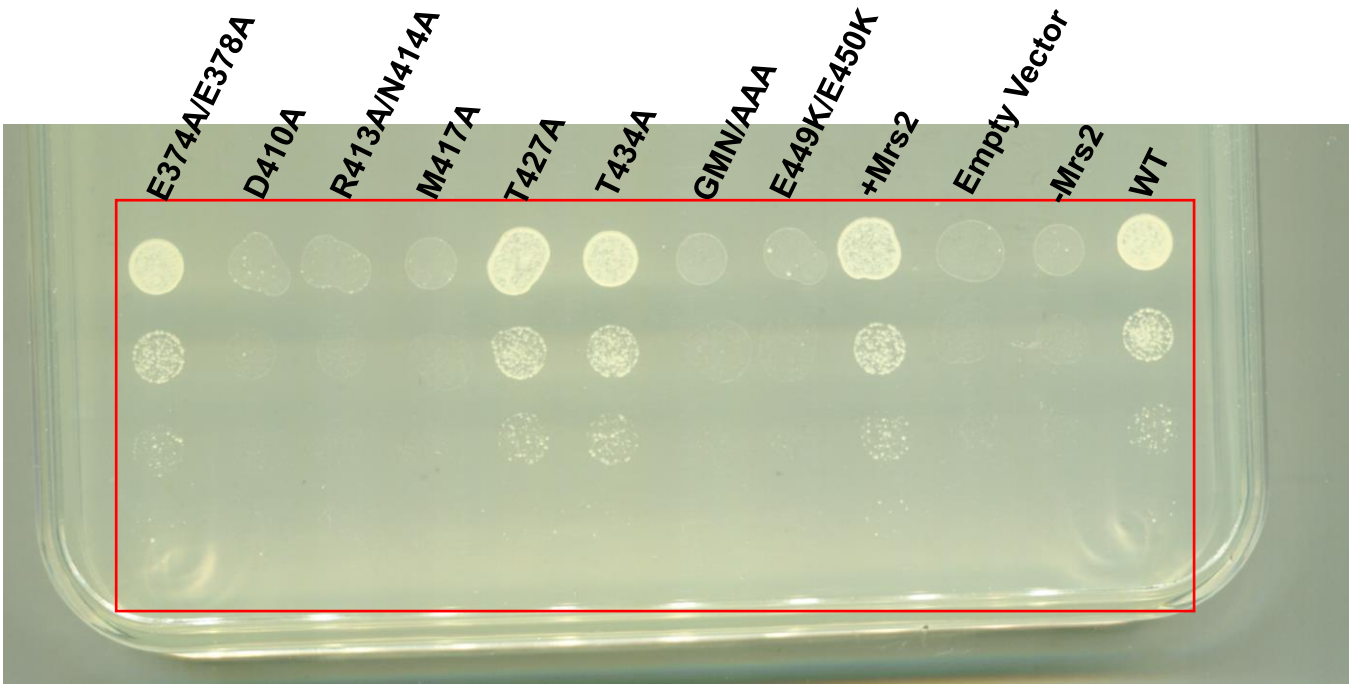

**Fig. 5a**

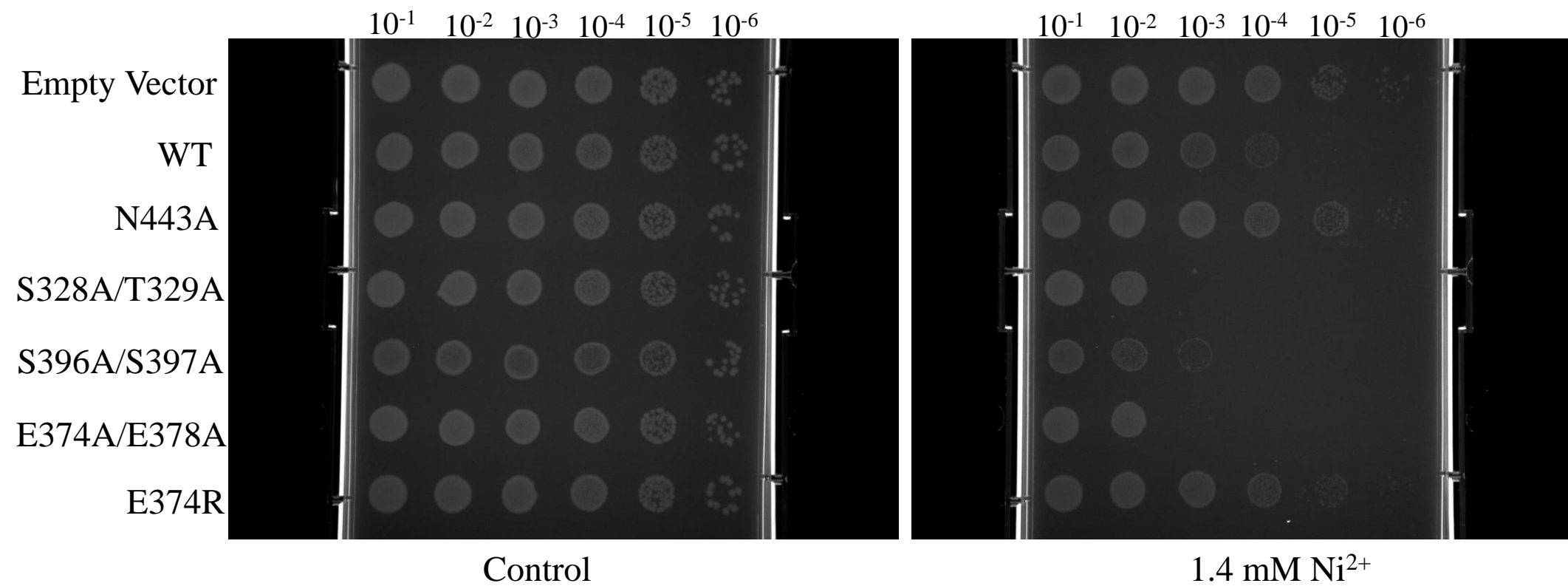

**Fig. 5b**

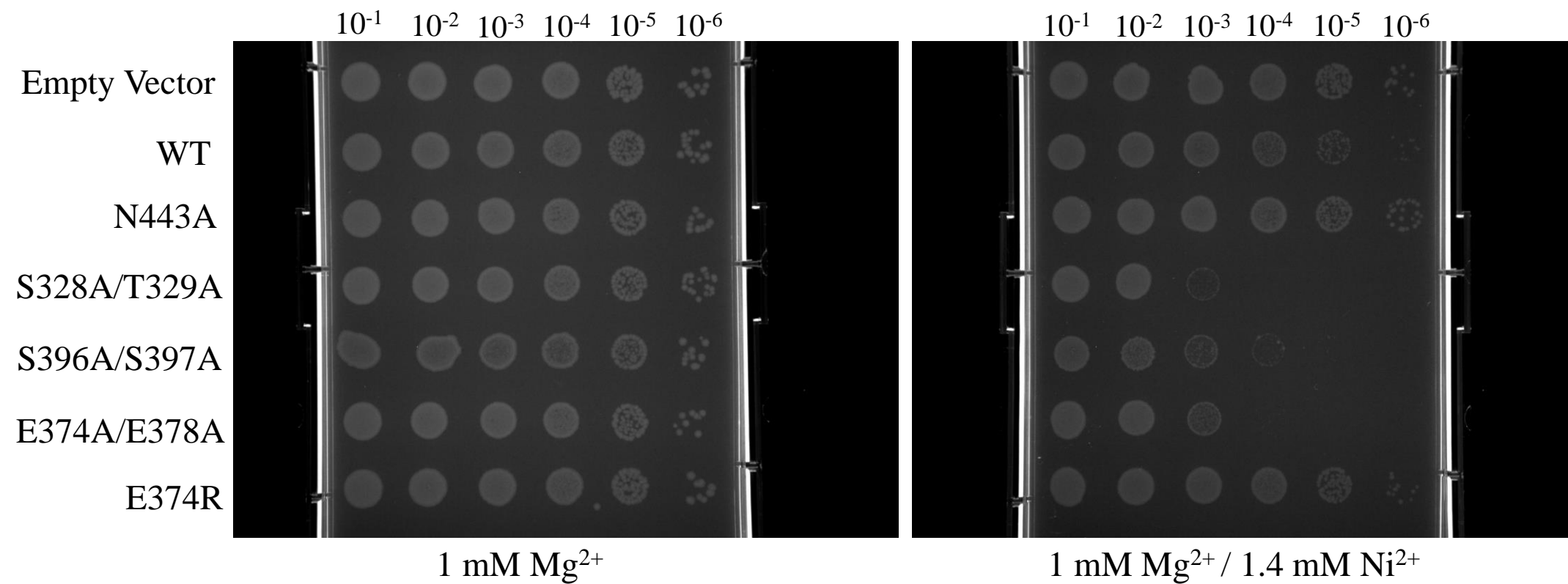

**Extended Data Fig. 1a**

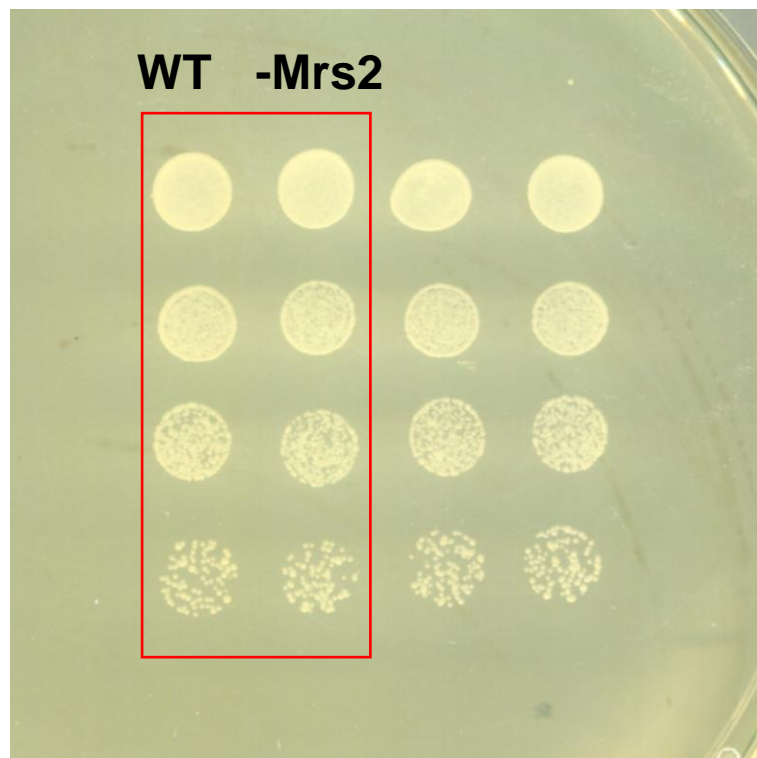

**YPD**

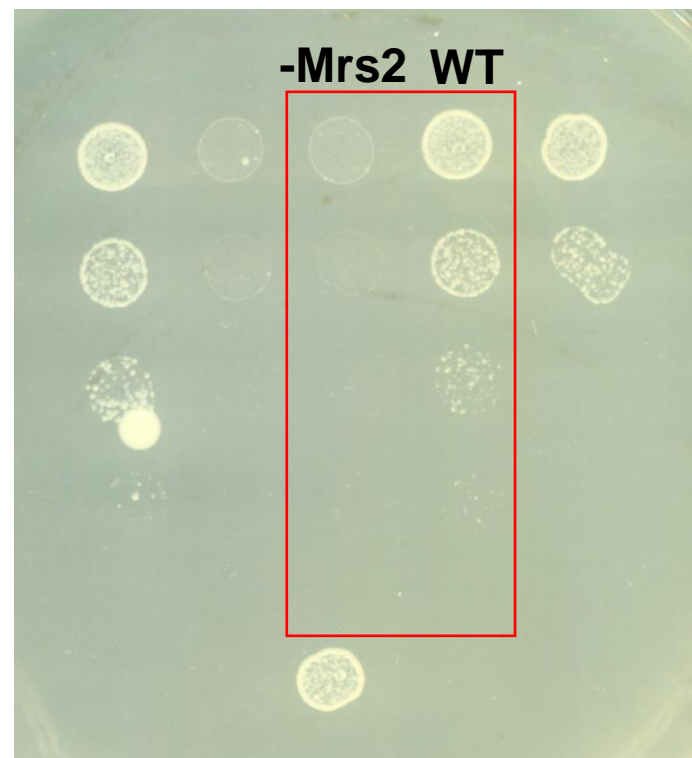

**YPG**

Supplement: Supplementary file 4 — Supplementary Source Data 1 [file 41594_2024_1432_MOESM4_ESM.pdf]

Source data for Fig. 1b

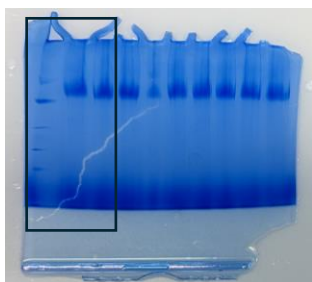

Supplement: Supplementary file 5 — Unprocessed native gel. [file 41594_2024_1432_MOESM5_ESM.pdf]

Source data for Fig. 5e

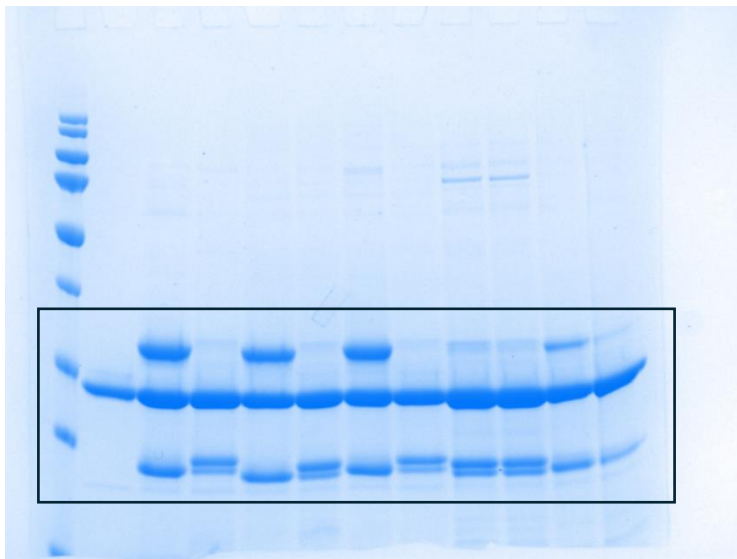

Trypsin

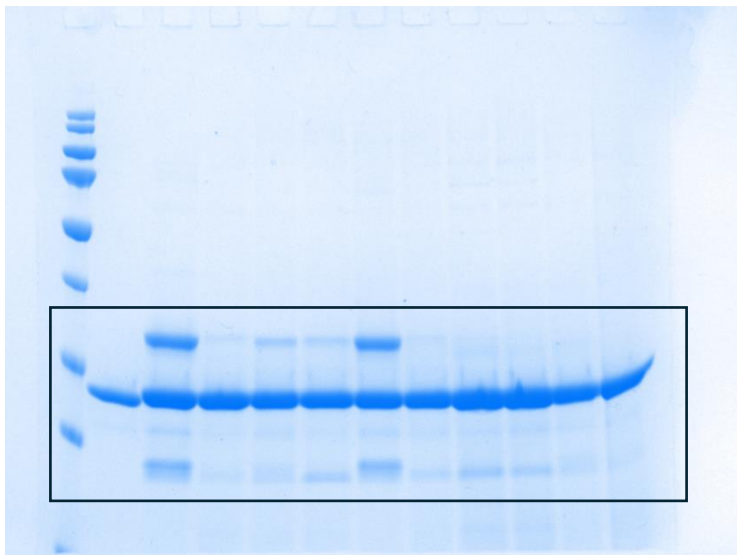

Chymotrypsin

Supplement: Supplementary file 7 — Unprocessed SDS–PAGE gel. [file 41594_2024_1432_MOESM7_ESM.pdf]

Source data for Extended Data Fig. 8b

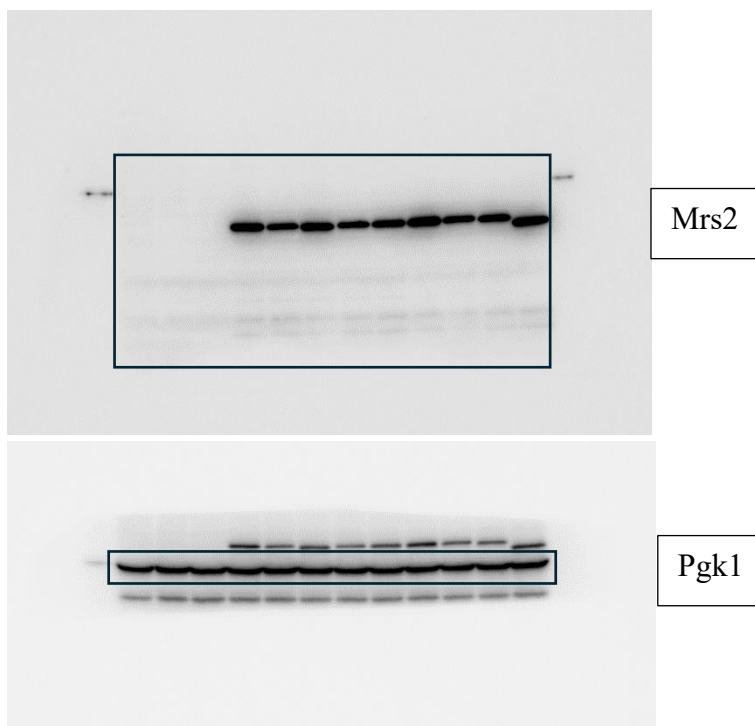

Supplement: Supplementary file 9 — Unprocessed western blots. [file 41594_2024_1432_MOESM9_ESM.pdf]
